# Lymphangiome du pied: un cas exceptionnel

**DOI:** 10.11604/pamj.2015.20.369.6714

**Published:** 2015-04-15

**Authors:** Youness Sasbou, Mustafa Nkaoui

**Affiliations:** 1 Service de Traumatologie-Orthopédie, CHU Ibn Sina, Rabat, Maroc

**Keywords:** Lymphangiome, kystique, pied, Lymphangioma, cystic, foot

## Image en medicine

Les lymphangiomes, plus récemment appelés malformations lymphatiques kystiques, sont des malformations lymphatiques matures, hémodynamiquement inactives, constituées de vaisseaux lymphatiques anormaux et de kystes de taille et de formes variées; Nous rapportons ici le cas d'une femme âgée de 30 ans ayant une malformation lymphatique macrokystique localisée au niveau du pied droit. Suivie depuis l'âge de 10 mois pour une tuméfaction congénitale sous-cutanée, indolore et molle du pied droit. L'échographie des parties molles, ainsi que l'imagerie par résonance magnétique (IRM), permettaient de retenir le diagnostic de malformation lymphatique macrokystique. Une exérèse chirurgicale en plusieurs temps a permis de réduire le volume tumoral en premier temps puis de reséquer l'ensemble de la tumeur en deuxième temps. Les suites opératoires étaient sans particularité et une récidive de la tumeur a été détecté après 6 mois. Les malformations lymphatiques macro kystiques sont localisées au cou dans 75 % des cas et aux creux axillaires dans 20 % des cas. L'atteinte du membre inferieur et particulièrement du pied est exceptionnelle.

**Figure 1:**
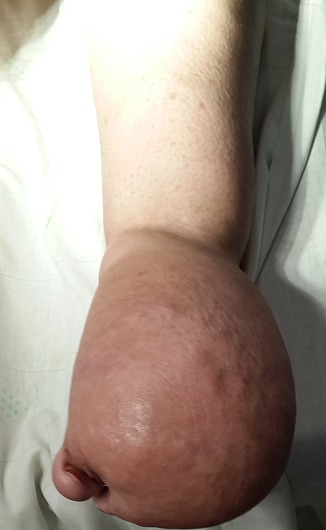
Lymphangiome du pied

